# SELF-REGULATION AND SELF-EFFICACY OF EXERCISERS DURING COVID-19

**DOI:** 10.1093/geroni/igad104.2308

**Published:** 2023-12-21

**Authors:** Candace Brown, Kira Chiles

**Affiliations:** University of North Carolina Charlotte, Charlotte, North Carolina, United States; University of North Carolina, Charlotte, Charlotte, North Carolina, United States

## Abstract

Employment, which was greatly impacted by the COVID-19 pandemic, caused alterations to the ‘work-from-home’ culture, and resulted in increased time sitting and decreased time in leisure activities, like exercise. This study explored the relationship of full time employment, intrinsic motivation, and self-efficacy to exercise with the understanding that social distancing and quarantining led to increased social isolation among all ages around the world. Methods Guided by the Self-determination theory, online data was collected from U.S. exercisers ages 18 and older who completed the Exercise Self-Regulation Questionnaire and Self-Efficacy for Exercise Scale during the height of the pandemic, in April 2020, and after, in February 2022. Results There were n = 320 participants that completed the surveys at T1 and n = 107 returned for T2 for a total of N = 427 participants. MANOVA results revealed a significance in intrinsic motivation (p < .001) in 2020 to exercise when gyms and outdoor spaces were closed. The greatest determinants of not achieving self-efficacy to exercise in 2022, when gyms and outdoor spaces were reopened, included “being bored” (36.1%) and “not enjoying” (29.6%) the activity. Conclusion Results suggest that full time employees, regardless of age, endorse high levels of personal accepted values and goals to exercise that are enjoyable, to them, for continued self-efficacy to exercise.

